# FDG PET-CT in Clinical Management of a Rare Case of Primary Hepatic Lymphoma: Role and Challenges

**DOI:** 10.1055/s-0043-1774734

**Published:** 2023-12-04

**Authors:** Tarun Kumar Jain, Naveen Gupta, Hemant Malhotra, Lalit Mohan Sharma

**Affiliations:** 1 Department of Nuclear Medicine, Mahatma Gandhi Medical College and Hospital, Jaipur, Rajasthan, India; 2 Department of Medical Oncology, Mahatma Gandhi Medical College and Hospital, Jaipur, Rajasthan, India

**Keywords:** primary hepatic lymphomas, FDG PET-CT, response assessment, Deauville score, SUVmax

## Abstract

The common differential diagnoses for multiple space-occupying hepatic lesions (SOL) are metastases, multifocal hepatocellular carcinoma, and abscess. Primary hepatic lymphomas are rare entities that present many challenges with regard to their management. Fluorodeoxyglucose positron emission tomography-computed tomography is extensively used for the staging and response assessment of lymphomas but it can be challenging and difficult to interpret in cases with isolated liver involvement. We hereby present the case of an 82-year-old lady who presented with multiple liver SOL.

## Introduction

Primary lymphoma of the liver is a rare entity characterized by isolated liver involvement and no involvement of any other sites until at least 6 months after diagnosis. It is important to recognize the entity as there are several unique management issues involving diagnosis, staging, treatment, and response assessment. We hereby discuss a case of an elderly lady with primary hepatic lymphoma (PHL) and role of Fluorodeoxyglucose positron emission tomography-computed tomography (FDG PET-CT) in its clinical management.

## Case Summary

**Fig. 1:**
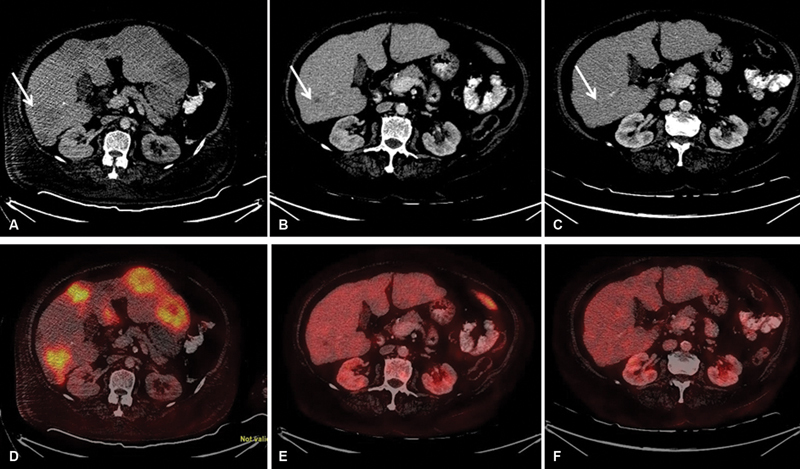
Transaxial contrast-enhanced computed tomography and positron emission tomography-computed tomographic images at baseline ( **A** , **B** ), interim ( **C** , **D** ), and end of treatment ( **E** , **F** ). Baseline images ( **A** , **B** ; *arrows* ) showing fluorodeoxyglucose (FDG) avid multiple heterogeneously enhancing discrete and coalescing soft tissue density hypodense lesions in both lobes of the enlarged liver parenchyma, with maximum standardized uptake value (SUVmax) values ranging from 11.2 to 19.3. In interim images ( **C** , **D** ), these lesions become low grade to non-FDG avid nonenhancing hypodense lesions and highest SUVmax was 2.6 versus 19.3; and end of treatment ( **E, F** ) images reveal non-FDG avid tiny ill-defined hypodensities in the segment IV, VI, and II of the liver parenchyma.

**Fig. 2:**
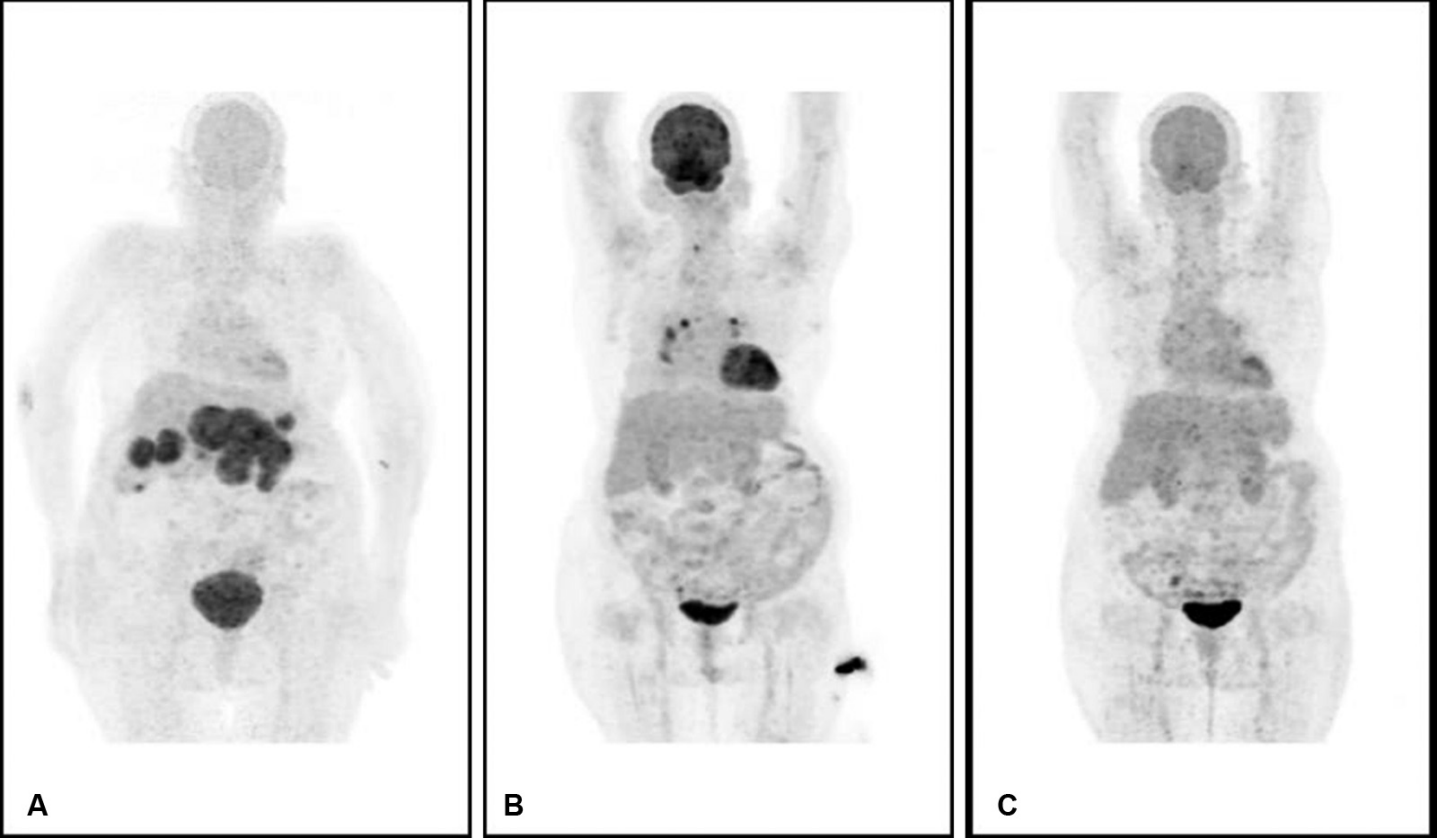
Maximum intensity projection positron emission tomography (PET) images of an 82-year-old lady with primary hepatic diffuse large B-cell lymphoma, at baseline ( **A** ), interim ( **B** ), and end of therapy ( **C** ). Baseline PET ( **A** ) showing multiple variable sized focal hot spots in the mid-abdominal region, while in interim PET ( **B** ) these abdominal hot spots disappear and few midline mediastinal and right supraclavicular hot spots appear and at the end of the therapy PET( **C** ) shows no hot spot in the entire body.

An 82-year-old lady presented with complaints of fatigue, fever, and shortness of breath. She had the following comorbidities: type 2 diabetes, hypertension, hypothyroidism, and interstitial lung disease. On clinical examination, positive findings were presence of crepitations in bilateral basal lung fields and liver palpable 3 cm below the right costal margin with a span of 18 cm. Performance status as per Eastern Cooperative Oncology Group score was 3. Blood investigation reports were as follows: hemoglobin, 10.4 g/dL (normal range: 12–16 g/dL); total leukocyte counts, 8,700/mm ^3^ (normal range: 3,500–10,000/mm ^3^ ); differential leucocyte count—neutrophils, 64%/lymphocytes, 28%/monocytes, 8%; platelet counts, 1,96,000/mm ^3^ (normal range: 1,50,000–4,50,000/m ^3^ ); peripheral blood film was unremarkable. Erythrocyte sedimentation rate was 18 mm per hour. Renal function and liver function tests were within normal limits. Human immunodeficiency virus (HIV), hepatitis B surface antigen, and anti-hepatitis C virus were negative during the lab examination. Ultrasonography of the abdomen showed presence of hepatomegaly with multiple round to oval variably sized hypodense lesions in both lobes of liver; largest lesion was measuring 6 × 5.2 cm in the left lobe. Ultrasound-guided biopsy was done from the liver lesion that showed diffuse infiltration with large, atypical cells with basophilic cytoplasm, round nuclei, vesicular chromatin, and prominent nucleoli with mitotic figures and karyorrhexis. On immunohistochemistry, these large cells showed CD20 positivity, along with BCL6 and MUM-1 positivity and negative for CD10. MIB-1 labelling index was 75 to 80%. These findings were suggestive of diffuse large B cell lymphoma, germinal center subtype. For staging purpose, whole body ^18^ F-FDG PET-CT was done. Contrast-enhanced CT and fused PET-CT images ( Fig. 1A,B ) reveal FDG avid multiple heterogeneously enhancing discrete and coalescing soft tissue density hypodense lesions in both lobes of the enlarged liver parenchyma. These lesions were markedly hypermetabolic, with maximum standardized uptake value (SUVmax) values ranging from 11.2 to 19.3. No other FDG-avid lesions were detected elsewhere, and marrow uptake was normal. The patient was treated with the following chemoimmunotherapy: rituximab, 375 mg/m ^2^ with cyclophosphamide, 400 mg/m ^2^ ; vincristine 1 mg; and oral prednisolone 60 mg for 5 days; cycle duration was 21 days. After two cycles, etoposide was added to this regimen at 50 mg on day 1 and 100 mg on days 2 and 3. Patient tolerated chemotherapy well and response assessment by interim whole-body PET-CT was done after four cycles. PET-CT ( Fig. 1C,D ) showed low-grade to non-FDG-avid nonenhancing hypodense lesions in the both lobe of liver parenchyma (largest in left lobe of liver SUVmax 2.6 vs. 19.3; ∼ 1.8 × 4.9 cm vs. 6.3 × 13.5 cm) and in compared to previous baseline PET-CT scan, significant decrease in FDG avidity and size of the liver lesion was noted. Additionally, there were some incidental findings in form of mild-to-moderate FDG avid mediastinal and right supraclavicular lymph nodes, which were reactive and proven by fine needle aspiration from the supraclavicular lymph node so estimated Deauville score was more likely 2 than 3. Further, three cycles of therapy were given and end of treatment PET-CT ( Fig. 1E,F ) showed non-FDG avid tiny ill-defined hypodensities in the segment IV, VI, and II of the liver parenchyma and compared to I PET-CT scan resolution of FDG uptake and decreased in size of lesions was noted and Deauville score was more likely 1. The comparative analysis of all three PET-CT scans is shown in Figs. 1 and 2 . The patient was followed up clinically every month for 6 months and remained asymptomatic.

## Discussion

PHLs are exceedingly rare entities and are defined by isolated liver involvement and no involvement of lymph nodes, spleen, bone marrow, or other tissues until at least 6 months after diagnosis. 1 They are frequently seen in the setting of chronic viral infections like hepatitis B, hepatitis C, and HIV or long-standing immunosuppression. 2 The most common histologic subtype seen in PHLs is a diffuse large B cell lymphoma. Infrequent subtypes are Burkitt lymphoma, T-cell lymphoma, or Hodgkin lymphoma. The patterns of involvement may be either solitary lesion, multifocal disease, or diffuse involvement. 3 4 5 ^18^ F-FDG PET-CT is an established imaging modality to investigate the disease extent in diffuse large B cell lymphoma and it is also helpful in response assessment with the help of Deauville scoring. There are only few case reports in the published literature that showed utility of ^18^ F-FDG PET-CT in staging and response assessment in isolated PHL. 6 7 This is in contrast with the known utility of ^18^ F-FDG PET-CT in the imaging of extranodal lymphomas, both in staging and response assessment. 8 9 Deauville scoring itself may have limitations since the liver itself is the primary site of involvement; however, mediastinal blood pool activity can be used for scoring grossly as done in present case.

The present case describes the use of FDG PET-CT in showing disease distribution and complete remission of PHL. The various treatment options described in literature are either combination chemotherapy or combination of surgery with chemotherapy. Theses modalities have been reported with good responses in the few reported cases. The optimal chemotherapy regimen is not defined but must be titrated according to the histologic subtype, patient's age, comorbidities, and overall fitness. 10 In present case, ^18^ F-FDG PET-CT was valuable by excluding other sites of involvement and showing the primary multifocal hepatic non-Hodgkin's lymphoma. The post-treatment interim FDG PET-CT showed markedly decreased in FDG uptake and size of the liver lesions and completion of PET-CT demonstrate nonmetabolic ill-defined hypodensities in the liver. On follow-up of 6 months, the patient remains asymptomatic.

## Conclusion

Presentation of lymphoma as isolated hepatic lesions is extremely rare. ^18^ F-FDG PET-CT is a valuable tool in establishing the extent and staging of lymphomas, and in our case, it was helpful in confirming the solitary involvement of the liver. However, imaging response assessment on FDG PET-CT is not so straightforward as accurate 5-point Deauville score and ratio Deauville score both cannot be ascertained owing to diseased liver parenchyma. In such a setting, the mediastinal blood pool may be taken as a point of reference to assess response to treatment.

## References

[1] Padhan R K Das P Primary hepatic lymphoma Trop Gastroenterol 2015 36 01 14 20 26591949 10.7869/tg.239

[2] Mrabet S Zaghouani H Mestiri S Akkari I Ben Jazia E Primary hepatic lymphoma in a patient with chronic hepatitis B Case Rep Gastroenterol 2020 14 03 632 636 33442342 10.1159/000511248 PMC7772829

[3] Chan W K Tse E W Fan Y S Zhang J Kwong Y L Khong P L Positron emission tomography/computed tomography in the diagnosis of multifocal primary hepatic lymphoma J Clin Oncol 2008 26 33 5479 5480 18955442 10.1200/JCO.2008.18.5413

[4] Mahajan S Kalra S Chawla M Dougall P Detection of diffuse infiltrative primary hepatic lymphoma on FDG PET-CT: hallmarks of hepatic superscan World J Nucl Med 2016 15 02 142 144 27134567 10.4103/1450-1147.167581 PMC4809157

[5] Pan B Wang C S Han J K Zhan L F Ni M Xu S C ^18^ F-fluorodeoxyglucose PET/CT findings of a solitary primary hepatic lymphoma: a case report World J Gastroenterol 2012 18 48 7409 7412 23326154 10.3748/wjg.v18.i48.7409 PMC3544051

[6] Seshadri N Ananthasivan R Kavindran R Srikanth G Chandra S Primary hepatic (extranodal) lymphoma: utility of [(18)F]fluorodeoxyglucose-PET/CT Cancer Imaging 2010 10 01 194 197 20926362 10.1102/1470-7330.2010.0028 PMC2999408

[7] Albano D Giubbini R Bertagna F 18F-FDG PET/CT and primary hepatic MALT: a case series Abdom Radiol (NY) 2016 41 10 1956 1959 27259334 10.1007/s00261-016-0800-1

[8] Othman A IA Nasr M Abdel-Kawi M Beyond lymph nodes: 18F-FDG PET/CT in detection of unusual sites of extranodal lymphoma Egypt J Radiol Nucl Med 2019 50 29

[9] Elhamady H Y Mostafa H M Elsayed H F Deauville score versus ratio Deauville score in the interpretation of interim 18F-FDG PET-CT and in prediction of outcome in children with FDG-avid extra-nodal lymphomas Egypt J Radiol Nucl Med 2022 53 217

[10] Mastoraki A Stefanou M I Chatzoglou E Primary hepatic lymphoma: dilemmas in diagnostic approach and therapeutic management Indian J Hematol Blood Transfus 2014 30 03 150 154 25114399 10.1007/s12288-013-0263-2 PMC4115079

